# An autosomal recessive mutation in *SCL24A4* causing enamel hypoplasia in Samoyed and its relationship to breed-wide genetic diversity

**DOI:** 10.1186/s40575-017-0049-1

**Published:** 2017-11-22

**Authors:** Niels C. Pedersen, Bonnie Shope, Hongwei Liu

**Affiliations:** 1 Center for Companion Animal Health, University of California, One Shields Avenue, Davis, CA 95616 USA; 2 Veterinary Dental Services, LLC, 530 Massachusetts Ave, Boxborough, MA 01719 USA; 3 Department of Veterinary Pathology, Microbiology and Immunology, University of California, One Shields Avenue, Davis, CA 65616 USA

**Keywords:** *Canis familiaris*, Samoyed, Familial enamel hypoplasia, *SLC24A4*, Short tandem repeats (STRs), Genetic diversity

## Abstract

**Background:**

Pure breeding of dogs has led to over 700 heritable disorders, of which almost 300 are Mendelian in nature. Seventy percent of the characterized mutations have an autosomal recessive mode of inheritance, indicative of positive selection during bouts of inbreeding primarily for new desired conformational traits. Samoyed suffer from several common complex genetic disorders, but up to this time only two X-linked and one autosomal dominant disorder have been identified. Previous studies based on pedigrees and SNP arrays have concluded that Samoyed breeders have done a good job in maintaining genetic diversity and avoiding excessive inbreeding. This may explain why autosomal recessive disorders have not occurred to the extent observed in many other breeds. However, an enamel hypoplasia analogous to a form of autosomal recessive amelogenesis imperfecta (ARAI) in humans has been recently characterized in Samoyed, although the causative mutation appears to have existed for three or more decades. The rise of such a mutation indicates that bouts of inbreeding for desired conformational traits are still occurring despite an old and well-defined breed standard. Therefore, the present study has two objectives: 1) measure genetic diversity in the breed using DNA and short tandem repeats (STR), and 2) identify the exact mutation responsible for enamel hypoplasia in the breed, possible explanations for its recent spread, and the effect of eliminating the mutation on existing genetic diversity.

**Results:**

The recent discovery of an autosomal recessive amelogenesis imperfecta (ARAI) in Samoyed provides an opportunity to study the mutation as well as genetic factors that favored its occurrence and subsequent spread. The first step in the study was to use 33 short tandem repeat (STR) loci on 25/38 autosomes and seven STRs across the dog leukocyte antigen (DLA) class I and II regions on CFA12 to determine the DNA-based genetic profile of 182 individuals from North America, Europe and Australia. Samoyed from the three continents constituted a single breed with only slight genetic differences. Breed-wide genetic diversity was low, most likely from a small founder population and subsequent artificial genetic bottlenecks. Two alleles at each autosome locus occurred in 70–95% of the dogs and 54% of alleles were homozygous. The number of DLA class I and II haplotypes was also low and three class I and two class II haplotypes occurred in 80–90% of individuals. Therefore, most Samoyed belong to two lines, with most dogs possessing a minority of existing genetic diversity and a minority of dogs containing a majority of diversity. Although contemporary Samoyed lack genetic diversity, the bulk of parents are as unrelated as possible with smaller subpopulations either more inbred or outbred than the total population. A familial disorder manifested by hypocalcification of enamel has been recently identified. A genome wide association study (GWAS) on seven affected and five unrelated healthy dogs pointed to a region of extended homozygosity on *Canis familiaris* autosome 8 (CFA8). The region contained a gene in the solute carrier 24 family (*SCL24A4)* that encodes a protein involved in potassium dependent sodium/calcium exchange and transport. Mutations in this gene were recently found to cause a similar type of enamel hypoplasia in people. Sequencing of this candidate gene revealed a 21 bp duplication in exon 17. A test for the duplication was in concordance with the disease phenotype. The exact incidence of affected dogs is unknown, but 12% of the 168 healthy dogs tested were heterozygous for the mutation. This population was biased toward close relatives, so a liberal estimate of the incidence of affected dogs in the breed would be around 3.6/1000. Theoretical calculations based on the comparison of the whole population with a population devoid of carriers indicated that eliminating the trait would not affect existing genetic diversity at this time.

**Conclusions:**

The contemporary Samoyed, like many other breeds, has retained only a small portion of the genetic diversity that exists among all dogs. This limited genetic diversity along with positive genetic selection for desirable traits has led to at least three simple non-recessive genetic disorders and a low incidence of complex genetic traits such as autoimmune disease and hip dysplasia. Unlike many other pure breeds, the Samoyed has been spared the spate of deleterious autosomal recessive traits that have plagued many other pure breeds. However, ARAI due to a mutation in the SCL24A4 gene has apparently existed in the breed for several decades but is being increasingly diagnosed. The increase in diseased dogs is most likely due to a period of intensified positive selection for some desired conformational trait. A genetic test has been developed for identifying the mutation carriers which will enable the breeders to eliminate enamel hypoplasia in Samoyed by selective breeding and it appears that this mutation can be eliminated now without loss of genetic diversity.

## Plain English summary

Samoyed evolved in the later part of the 19^th^ century out of interest and concern for the dogs that accompanied expeditions to the Arctic and Antarctic. Their ancestry is from indigenous Laika of Siberia and Russia. Samoyed are now of average popularity but have seen significant population declines during WWI and a marked population increase in the 1980s and 1990s in the UK followed by an even more precipitous decline to present. Although previous pedigree and SNP-based testing suggest that the breed is not in any immediate danger, the present study indicates that genetic diversity may not be as great as presumed and that inbreeding is still a concern. Lack of genetic diversity coupled with bouts of inbreeding has led to a high incidence of complex genetic disorders and a high proportion of deleterious mutations with a recessive mode of inheritance in most dog breeds. Samoyed do suffer from several complex genetic disorders and two simple X-linked and one dominant conditions, but Samoyed have been surprisingly spared the autosomal recessive disorders that have plagued other breeds. However, a dental condition known as enamel hypoplasia has been recently recognized in the breed and although the causative mutation has apparently existed for many generations, the disorder is being diagnosed with increasing frequency. The incidence of carriers of the mutation appears to be low, which should allow breeders to eliminate the trait from the total population without further loss of genetic diversity. However, Samoyed breeders must take care that no further genetic diversity is lost and be prepared for other deleterious autosomal recessive traits that can result from conformation-driven bouts of strong human-directed positive selection. Pedigree derived breeding information should also be confirmed and augmented by DNA-based testing.

## Background

### History of Samoyed breed

**Fig. 1:**
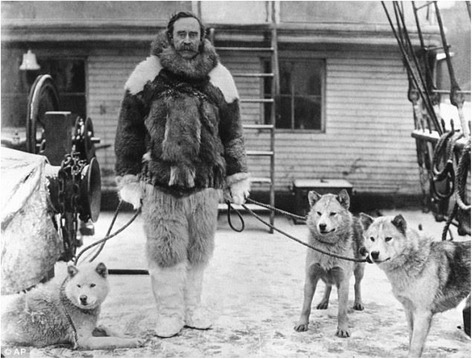
Robert Peary Sr in Greenland with some of the sledge dogs used in Greenland. Source: Willy Ley The Poles New York, New York: Time Incorporated, 1962. These dogs were of the Samoyede type. However, large heavy coated dogs of varied appearance were also used. Ancestors of such dogs (Samoyede or Samoyed) became more known for their gentle demeanor around people, alert and active personalities, striking thick double coats and noteworthy solid or patterned white, biscuit or cream colors than for pulling sledges

The Samoyed is considered one of the basal breeds developed in the late Victorian era from indigenous Laika used by the Nenets (Samoyede) peoples of Northwest Russia and Siberia. Like many pure breeds, Samoyed have a convoluted history with several versions [1].^1^^,^ 2 ^,^ 3 ^,^ 4 ^,^ 5 ^,^ 6 Samoyed belong to what is known as the Arctic or Nordic group that include the Alaskan Malamute, Chow Chow, German shepherd dog and several other Spitz-type breeds. The peoples of the arctic regions have kept dogs over millennia and selectively bred dogs that were most helpful to them for hunting, guarding camps and villages, pulling sledges and carrying packs, companionship and even sharing the hearth and bed. Dogs of this utilitarian type first appeared in Russian dog shows in the late 19^th^ century.^7^ Robert Peary was the first westerner to use dogs to pull sledges during his expeditions to Greenland in 1891–92.^8^ Sledge dogs were first brought keenly to the attention of the Western world by Fridtjof Nansen, a Norwegian explorer who used teams of “Samoyedes” as sledge dogs during his 1894 expedition to the North Pole [2]^8^ Fig. 1.

The rise of the Samoyed in America and other countries was preceded in the UK by the activities of Sir Ernest Kilburn-Scott and his wife Clara.^1–5,^ 9 The first foundation dog for the breed in England was a brown and white dog named Sabarka that was purchased by Ernest in Archangel, Russia in 1889 as a gift for Clara.^9^ However, it was the white dogs that he encountered in his travels that caught his fancy and led them to purchase a cream-colored bitch named Whitey Petchora [1]. The offspring of these two dogs were bred by the Kilburn-Scott’s and others and helped popularize the breed. During their stay in Australia from 1907 to 1910, the Kilburn-Scott’s became involved in the plight of former sledge dogs returning from Antarctic explorations. Returning dogs were sometimes returned to sledge-dog breeding kennels in South New Zealand, but most were subjected to strict, long-term, expensive and therefore fatal quarantines in New Zealand and some were exhibited in zoos in New Zealand and Australia. Dogs saved from such conditions and environments were a common source of breeding stock for evolving Arctic-type breeds during the late 19th and early 20th centuries. Ernest and Clara brought several of these dogs back with them from Australia in 1910, including a large white dog named Antarctic Buck, which they acquired from the Sydney zoo [1]. Antarctic Buck died of canine distemper after coming to the UK but at least five of his progeny survived and were integrated into the Samoyede in the UK.

The Kilburn-Scott’s showed their dogs in the foreign dog classes in the UK for several years and the breed was given conditional registration in 1902 and formal recognition under the original name Samoyede in 1912 by the UK kennel club. The name was later changed to Samoyed by both the AKC and UKC. The American connection was stimulated when a member of a famous European royal family, Rose de Mercy-Argenteau (Princess de Montglyon), was smitten with a large white champion Russian Samoyed named Moustan entered into the 1902 St. Petersburg dog show by Grand Duke Michael of Russia.^7^ Moustan was given to the countess as a gift and brought along with three other Samoyed to the USA in 1904. Moustan was shown extensively in America and the breed recognized by the AKC in 1906.

### Changes in Samoyed population size over time

The Samoyed breed has retained an average popularity compared to other breeds over the century and registration numbers have been relatively steady in both the UK and USA except for a brief decline during WWI and a sharp rise after 1980 to a peak of 1200 registrants in the UK in 1995 and decline to pre-peak levels of 300 by 2014.^10^ The Samoyed currently ranks 64th in popularity in the USA with a steady 1500 new registrations each year per AKC records.^4^

### Known or presumed heritable diseases of Samoyed

Several diseases that appear to be heritable, but not genetically defined, have been observed at low frequency in the breed.^11^^,^ 12 ^,^ 13 Many of these disorders have evolved with the domestic dog over time and inherited by descent as breeds have been created [3]. Except for hip dysplasia, which is considered one of the more serious disorders of Samoyed, most heritable and potentially heritable disease traits of the breed have been of minor importance.^11^ There are only three simple deleterious genetic disorders in Samoyed with defined causes, X-linked glomerulopathy [4], X-linked progressive retinal atrophy [5], and an incomplete dominant short-limbed defect with ocular abnormalities [6, 7].

Except for two simple X-linked and dominant genetic disorders, Samoyed have been surprisingly free of deleterious autosomal recessive traits, which are frequent indicators of artificial genetic bottlenecks and popular sire effects and the bane of many other breeds. Over three hundred heritable disorders not linked to any breed standard were identified among the top 50 pure breeds of dogs as of 2010 [8] and many more have been reported since that time. Eighty of these disorders have proven genetic causes and 71% of these are due to autosomal recessive mutations, with the German shepherd and Golden retriever having the highest number [8]. Deleterious traits associated with autosomal recessive mutations are often not recognized until the incidence of affected dogs reaches levels of concern (i.e., livability and/or treatability) and age at onset. Twenty percent of dogs will carry a deleterious autosomal recessive trait by the time the incidence of actual disease reaches 1 %.

Deleterious autosomal recessive mutations occur with some frequency in large populations in nature, but they either remain at low frequency or eventually disappear. However, simple autosomal recessive mutations can rapidly amplify in small populations (such as dog breeds) if the defective allele is linked to regions of the genome under strong positive selection. In the case of many pure breeds of dogs, this strong positive selection is human directed towards conformation traits that are extolled by the show ring [9, 10]. The rapid expansion of such deleterious traits in a pure breed of dogs is often aided by their recessive nature, late age of onset, small population size, and a lack of genetic diversity due to small founder populations and other artificial genetic bottlenecks. Although the Samoyed has escaped the problems with deleterious autosomal recessive diseases experienced by many other breeds, a type of ARAI has been recently appeared in the breed and is apparently increasing in incidence. The recognition of a deleterious autosomal recessive disease in the breed brings into question the status of genetic diversity that still exists and how that diversity is being distributed.

### How genetic diversity relates to deleterious genetic disorders

The amount of genetic diversity in contemporary dog breeds reflects several factors. One factor is the number of founder animals that went into creation of the prototypic Samoyeds before the breed was recognized and its registry closed to outside dogs. If the founder population is large and genetically diverse, the prototypic dogs that are selected as breed founders will reflect that diversity. Once the registry is closed to outside dogs, the amount of genetic diversity of the new pure breed cannot theoretically increase except by rare natural mutation. Therefore, the goal of proper pure breeding is to select only the healthiest dogs and maintain the original genetic diversity and health for the rest of the breed’s existence. This requires random or careful selection of sires and dams. A second factor involves a loss of genetic diversity after the registry has been closed. It has been calculated that the average pure breed of dog retains only 87% of its original diversity [11], which is probably a generous estimate. A certain amount of genetic diversity is lost through human-directed selection for specific desired traits, something that often occurs early on during what is called “breed refinement.” Breed refinement is the process of solidifying objectives of the breed standard and assuring that the desired traits will breed true regardless of the sires and dams that are chosen. Further loss of genetic diversity occurs from several forces, such as geographic isolation, catastrophic events such as world wars or famines, and most importantly, slow or rapid deliberate changes in breed appearance [12–16].

Positive selection for conformation traits has a much greater effect on genetic diversity than breeding for performance traits [9]. Performance traits have a lower heritability than conformation traits and are much less subject to changes in how a breed standard is interpreted. Performance requires that the basic form and structure of the early dog is maintained as much as possible, while conformational change is more esthetic than functional. Nonetheless, if breed standards and phenotype never changed, there would be no reason for inbreeding to amplify and solidify new traits. Breeders of purebred dogs in the UK have faced criticism over deviations from UK Kennel Club breed standards that have had a detrimental impact on health. This concern was impetus for a study of conformation-related disorders among the top 50 UK Kennel Club registered breeds using a novel index to score to determine how conformational changes related to health [10]. Each of the 50 breeds was found to have at least one aspect of its physical appearance that affected health and 84 disorders were either directly or indirectly associated with conformation. The miniature poodle, Bulldog, Pug and Basset hound had the highest incidence of disease conditions related to conformation. A more detailed study on the effect of severe phenotypic change on genetic diversity and health has been reported for the Bulldog [13].

There is no doubt that a show judge’s interpretation of ideal conformation affects how breeders select the parents of subsequent generations of puppies [9]. However, there are other factors that can affect the level of inbreeding and potential loss of genetic diversity. If the population of available bitches and stud dogs is small, and/or if breeders must also select against many health problems, the ability to find the healthiest and least unrelated parents at proximity can be difficult [16]. One of the greatest influences on inbreeding and genetic diversity is known as the “popular sire effect.” A show winning stud dog has the capability through his own offspring and offspring of sons and grandsons to produce far more offspring than a show winning bitch [16]. These various situations often require inbreeding as the fastest means to reach a genetic goal. However, inbreeding and loss of genetic diversity are not synonymous terms. Inbreeding can lead to a high degree of relatedness between individuals in a population, and can cause a loss of genetic variation when focused on particular lines, but if done with care most of the genetic variations present in the breed at its origin can be preserved [11]. The level of inbreeding can be determined by accurate pedigrees but genetic diversity is best identified by actual analysis of DNA. An example is the Standard Poodle, which has been admittedly inbred along a certain famous mid-century bloodline as based on pedigrees but still retains a great deal of genetic diversity when tested by DNA [14]. This is because a majority of the genetic diversity based on DNA analysis was contained in a minority of less desired outbred dogs, while a minority of genetic diversity was contained in the majority of the more desirable inbred dogs [14].

### A familial enamel hypoplasia newly recognized in Samoyed

Samoyed breeders have not recognized any deleterious traits caused by autosomal recessive mutations up to this time, which has been reassuring as most breeds suffer from several recessive disorders [8, 10]. However, Samoyed have been recently seen in increasing numbers by veterinary practitioners and veterinary dentistry specialists for abnormal and badly discolored teeth, irregular tooth surface, heavy tarter accumulation, gum disease, caries and tooth loss. This was initially dismissed, as have similar disorders in several other breeds, as being caused by some sort of environmental insult occurring during puppy-hood when the adult dentition was forming. One of the authors (BS) brought the disorder to the attention of the other authors (NCP and HL) because of their work with a familial enamel hypoplasia in Italian Greyhound [17]. This interaction led to the present study, which identified the genetic cause for this disorder and to develop a test to identify carriers of the trait. The discovery of a heretofore uncharacterized autosomal recessive disease in the breed highlights the propensity of pure breeding to amplify autosomal recessive mutations. A DNA-based knowledge of genetic diversity in their breed can both explain why such mutations occur and how they are amplified in the population. DNA testing can be also used to decide whether to retain the mutation but select against homozygotes or eliminate the trait by identifying and removing affected dogs and carriers from the breeding pool. If the recessive mutation is extremely common and genetic diversity low, eliminating the trait can lose valuable genetic variation.

## Methods

### Sample collection

The 182 Samoyed in the study were from the North America (North America = 144), Europe (*n* = 32) and Australia (*n* = 6). Fourteen dogs were clinically affected, including 11 from the USA and three from Europe. Samples were solicited through web communications and owners/breeders wishing to submit DNA for testing were asked to make contact and request a DNA test kit that contained 2–3 cytology brushes for each dog. Further DNA was required from some cases in order to do extra investigations, so blood samples were collected by the veterinarian looking after those cases. Owners were encouraged to list each dogs’ registration number, registered name, call name of sire and dam, age, sex (intact, spay, castrated), coat color, whether affected or not, existence of other health conditions, and relationship if known to affected dogs. Digital photographs of teeth and pedigrees were also requested whenever possible.

### DNA extraction

DNA was extracted from a single cytology brush by heating at 95 °C in 400 μl 50 mM NaOH for 10 min and the pH neutralized with 140 μl 1 M Tris–HCl, (pH 8.0) [18]. Blood samples (200 μl) were extracted using QIAGEN QIAamp®DNA blood mini and midi kits (QIAGEN Inc., Valencia CA, USA).

### Genetic diversity testing

Thirty-three STR loci from across the canine genome were multiplexed into two panels, one consisting of 20/21 di-STRs recommended for canine parentage verification by the International Society of Animal Genetics (ISAG)^13^ and a second consisting of two di-STRs and 10/15 tetra-STRs validated for forensic testing [19]. Amelogenin gene primers for gender determination were also included [20]. Primers, dye labels, repeat motif, allele size range and known alleles for this set of markers can be found in Pedersen et al. [21]. Genotyping was conducted by the Veterinary Genetics Laboratory (VGL), UC Davis, and data were analyzed using STRand software [22]. Examples of genetic diversity profiling of various breeds using these markers can be found at the VGL website.^14^

### Determination of DLA class I and II haplotypes

Four dinucleotide STRs from regions flanking the DLA class I (*DLA88*) and three STRs associated with DLA class II (DLA-DRB1, −DQA1, −DQB1) were identified on Dogset.^15^ Locus designations, primer sequences, number of alleles and allele size ranges have been previously published [14].

### Statistical analyses

Genetic diversity estimates were calculated from allele and allele frequency data from 33 genomic STR loci using GenAIEX 6.5 [23]. Principal coordinate analysis was also done with GenAIEX 6.5.

Internal relatedness (IR) reflects the relationship of an individual’s parents as described by Amos et al. [24] and based on an earlier calculation by Queller and Goodnight [25]. IR is a measure of heterozygosity that weights allele sharing by allele frequency and is highly correlated with standardized heterozygosity and with heterozygosity weighed by locus [26]. Based on internal testing, an IR value of ≥0.25 was found to equate to offspring of full-sibling parents. IR values were graphed in two manners: 1) comparing individual Samoyed with other Samoyed in the population, and 2) comparing the IR values of all Samoyed with the IR values of a large population of randomly breeding and genetically diverse village (indigenous) dogs from the Middle East, SE Asia and Pacific region [27]. The frequency of alleles at each STR locus is compared with the frequency of the same alleles at the same loci in the village dog population. The adjustment is called IR-village dog or IRVD and approximates the amount of diversity that was lost because of genetic bottlenecks that have occurred since the first Samoyed founders were selected and the registry closed to outside introgressions.

### Genome wide association study (GWAS)

Among the 182 Samoyed sampled, 7 dogs with enamel hypoplasia and 5 healthy dogs were selected for genome-wide association study (GWAS) based on clinical criteria described herein. SNP genotyping was performed at GeneSeek (Lincoln, NE) with the Illumina (San Diego, CA) CanineHD Genotyping BeadChip containing 230 K markers placed on the CanFam3 reference sequence and the results were analyzed using PLINK (Purcell et al. 2007). Data from GWAS was analyzed subjected to Bonferroni correction to account for multiple comparisons. The strongest signal from the unadjusted association analysis was termed *P*_raw_. The thresholds for final genome-wide significance was determined by MaxT permutation testing using 100,000 permutations with PLINK.

### Sequencing of *SLC24A4*

The genetic analysis of *SLC24A4* was conducted on genomic DNA from 4 Samoyed dogs (2 affected and 2 control). The complete sequence of *SLC24A4* is publicly available and can be found on chromosome 8 at positions 1,507,274–1,679,208.^16^ In this study the complete DNA sequence was analyzed. Primers were designed in the intronic regions flanking the exons and evaluated with Netprimer.^17^ Primers were tested for efficient product amplification on a 2720 Thermal Cycler (Applied Biosystem). Sequences and the amplicon size of each primer pair are shown in Table 1. The PCR for all exons was performed as follows: initial denaturation at 94 C for 3 min followed by 30 cycles as follows: 94 °C × 30 s, 61 °C × 30 s, 68 °C × 3 min. The PCR products were purified with the ExoSap (USB, Cleveland, OH) per the manufacturer’s recommendations and directly sequenced using the BigDye terminator Sequencing Kit v3.1 (Applied Biosystem/Life Technologies, Carlsbad, CA). The sequencing products were purified using Performa DTR Ultra 96-well plate kit (EdgeBio, USA) according to the manufacturer’s recommendations, and electrophoretically separated on an ABI 3730 DNA analyzer (Applied Biosystems/Life Technologies, Carlsbad, CA). Sequences were verified and aligned using the software Sequencer version 4.9.1 (Gene Codes Corp., Ann Arbor, MI).

**Table 1:** Primers designed for *SLC24A4* gene amplification and sequencing

Exon	Prod.(bp)	Forward primer 5′-3’	Reverse primer 5′-3’
1	908	CCAAATCAGGGAGCCTCAGAG	GTTCTTGCCTGGGCGTGAG
2	388	GTATCACTCCCTACAGGTGGCTCTA	TACCTTCCGTGCCTTACAGCC
3	641	GGTGGCTAATCCAGCGTTCG	GCATCATGGGAATCACGACAGT
4	529	CCGCTGCTGCTTAGAGATGC	GGAAGCACCTGTCCTCAGAAGC
5–7	1669	ACAGGGGACTCGGGCTGA	GTGCTGTGGGTTCTCTGGTCA
8	555	GTAGGCAGGTGCGTAGGGACA	AGGAGCACTGAGGAAACCATCTG
9–10	1916	GGGCTTCAGATGGCAGGATG	GGATGGCAGGTGAGAGTGAGG
11	648	TCAGGATGGGCTCAGCAAGAG	GGGGCAGGACTGGATTGAGG
12	617	GCAAACTTAGAGGGATGGTGAGG	TCAAACTACGGGTGATGTGTGG
13	506	GGGTGACATTATGGAGGTGTGTG	CCACACACCTCTGCTGAAACC
14	677	GCTGTCCTGAATCAGAACCTGG	GCTTTGTTCAATGTCACACTGGTG
15–16	1017	AGTCCTCTGTGGAGAGCAGCG	AACAATGGACGGGTCGGTG
17	599	GCAGCAGCCATGTGTCCTG	GAGGGCAGGGCTCCGAG
6 s^a^		GGCTGTGCTGAAACCCTGG	
16 s^a^		GCACTAGGCTCTGAGAACACTGC	

^a^6s and 16 s are additional primers required for sequencing of the large exons 6 and 16

To confirm the identified mutation as causative, all 182 Samoyed, which included those used for GWAS, were genotyped for a 21 bp duplication in exon 17. A PCR reaction with SLC24A4-F (FAM labeled) SLC24A4-R primers (Table 1) was performed using 2 mM Mg^2+^ at 95 °C for 5 min, and 85 °C for 5 min followed by 5 cycles of 94 °C × 1 min, 60 °C × 30 s, 72 °C × 30 s, and another 28 cycles of 94 °C × 45 s, 60 °C × 30 s, 72 °C × 30 s followed by 72 °C for 30 min using Taq DNA polymerase (Denville Scientific), and electrophoretically separated on an ABI DNA analyzer (Applied Biosystems). The predicted size of the wild type allele was 92 bp and 113 bp for the insertion mutant, which was verified using the software STRand [22].

## Results

### Assessment of genetic diversity based on 33 genomic STR markers

#### Standard genetic assessment

The purpose of this study was to utilize 33 STR loci on 25 canine autosomes to study the genetic background of a population of 182 Samoyed from various parts of the world as part of a study of a familial enamel hypoplasia. It was presumed that this population would identify over 95% of existing genetic diversity and heterogeneity in Samoyed based on experience with other breeds.^14^ Table 2 lists the alleles and allele frequency identified at each of the 33 genomic STR loci. This data was then used for a standard genetic assessment (Fixation indices) of the total population of 182 dogs (Table 3). The average number of alleles (Na) found at each locus was 6.09 (SE 0.357), while the average number of effective alleles (Ne) at each locus was 3.24. The Na was like a number of purebreds that have been studied, but the He was the lowest of any other breed studied to date by our group.^14^ These values indicated that genetic diversity was heavily influenced by a small number of founders.

**Table 2:** Allele designations and frequency at each of the 33 autosomal STR loci for 182 Samoyed

1. AHT121	2. AHT137	3. AHTH130	4. AHTh171-A	5. AHTh260	6. AHTk211	7. AHTk253	8. C22.279	9. FH2001	10. FH2054	11. FH2848	12. INRA21	13. INU005	14. INU030	15. INU055	16. LEI004	17. REN105L03	18. REN162C04	19. REN169D01	20. REN169O18	21. REN247M23	22. REN54P11	23. REN64E19	24. VGL0760	25. VGL0910	26. VGL1063	27. VGL1165	28. VGL1828	29. VGL2009	30. VGL2409	31. VGL2918	32. VGL3008	33. VGL3235
94 (0.286)	131 (0.058)	119 (0.192)	219 (0.437)	236 (0.157)	89 (0.335)	284 (0.030)	116 (0.280)	132 (0.019)	148 (0.011)	232 (0.003)	95 (0.937)	110 (0.154)	144 (0.19)	210 (0.005)	85 (0.25)	227 (0.096)	200 (0.027)	202 (0.437)	162 (0.104)	266 (0.096)	226 (0.129)	139 (0.456)	12 (0.124)	17 (0.245)	9 (0.005)	14 (0.173)	14 (0.008)	9 (0.192)	13 (0.033)	9 (0.003)	10 (0.003)	12(0.313)
96 (0.019)	133 (0.184)	121 (0.684)	225 (0.066)	238 (0.005)	91 (0.61)	286 (0.107)	118 (0.124)	136 (0.102)	152 (0.0035)	234 (0.286)	97 (0.047)	122 (0.426)	146 (0.005)	212 (0.005)	95 (0.75)	229 (0.173)	202 (0.19)	208 (0.030)	164 (0.206)	268 (0.349)	232 (0.58)	143 (0.33)	13 (0.173)	18 (0.069)	12 (0.596)	15 (0.140)	18 (0.005)	11 (0.184)	14 (0.129)	12 (0.011)	13 (0.277)	13(0.346)
100 (0.112)	145 (0.014)	127 (0.011)	227 (0.140)	242 (0.214)	93 (0.055)	288 (0.146)	120 (0.08)	140 (0.027)	156 (0.005)	236 (0.214)	101 (0.016)	124 (0.346)	148 (0.560)	214 (0.129)		231 (0.1)	204 (0.593)	212 (0.121)	166 (0.560)	270 (0.231)	234 (0.266)	145 (0.107)	14 (0.102)	19 (0.019)	13 (0.014)	17 (0.003)	19 (0.799)	12 (0.014)	15 (0.266)	13 (0.104)	14 (0.187)	14 (0.275)
102 (0.324)	147 (0.25)	129 (0.112)	229 (0.2)	246 (0.371)		290 (0.357)	122 (0.055)	144 (0.495)	160 (0.005)	238 (0.385)		126 (0.022)	150 (0.245)	216 (0.179)		233 (0.030)	206 (0.129)	216 (0.135)	168 (0.118)	272 (0.310)	236 (0.022)	147 (0.107)	18 (0.223)	20 (0.055)	14 (0.071)	19 (0.148)	20 (0.176)	13 (0.201)	16 (0.115)	14 (0.593)	15 (0.08)	15 (0.025)
106 (0.038)	151 (0.239)		235 (0.159)	250 (0.12)		292 (0.357)	124 (0.404)	148 (0.003)	168 (0.629)	240 (0.074)		132 (0.0522)		218 (0.681)		235 (0.003)	210 (0.060)	220 (0.058)	170 (0.011)	278 (0.014)	238 (0.003)		19 (0.022)	21 (0.324)	17 (0.283)	20 (0.003)	22 (0.008)	14 (0.382)	17 (0.247)	15 (0.247)	16 (0.052)	16 (0.033)
108 (0.115)	153 (0.255)			252 (0.143)		294 (0.003)	126 (0.005)	152 (0.091)	172 (0.157)	244 (0.038)						239 (0.055)		222 (0.008)					20 (0.066)	22 (0.179)	18 (0.030)	23 (0.003)	23 (0.003)	15 (0.027)	18 (0.206)	16 (0.041)	17 (0.209)	17 (0.008)
112 (0.036)							134 (0.052)	156 (0.264)	176 (0.165)							241 (0.527)		224 (0.206)					21 (0.231)	23 (0.049)		24 (0.22)			21 (0.003)		18 (0.041)	
114 (0.002)									180 (0.025)							245 (0.016)		226 (0.005)					22 (0.058)	24 (0.060)		26 (0.308)					19 (0.003)	
116 (0.066)																							26 (0.003)			31 (0.003)					20 (0.008)	
																															21 (0.030)	
																															22 (0.099)	
																															23 (0.011)

**Table 3:** Genetic assessment of Samoyed based on 33 genomic STR markers

Pop		N	Na	Ne	Ho	He	F
Total	Mean	182	6.091	3.237	0.615	0.641	0.041
	SE		0.357	0.209	0.026	0.027	0.010

The observed heterozygosity (Ho) for the 182 dogs was 0.615, which was somewhat lower than the expected heterozygosity (He) of 0.641 that would be anticipated for a population that is in Hardy-Weinberg equilibrium (i.e., total random selection of parents). The difference in Ho and He led to a fixation index (F) (a measure of inbreeding) of +0.041. This indicated that a small subpopulation of the 182 dogs was more inbred (less heterogeneous) than the total population.

#### Principal coordinate analysis (PCoA)

**Fig. 2:**
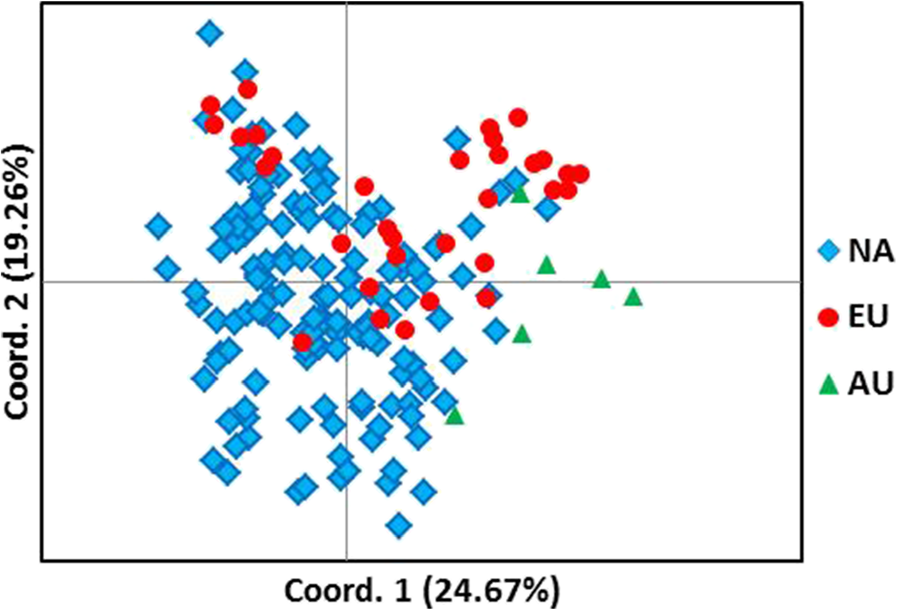
PCoA plot of 182 Samoyed from North America (116 US +28 Canada), Europe (*n* = 32) and Australia (*n* = 6) based on alleles and allele frequencies at 33 genomic STR loci

Principal coordinate analysis was used to approximate the relatedness of individuals in a population to each other using the allele frequency data obtained from the 33 autosomal STRs (Table 2). The actual results are in multiple dimensions forming a sphere, but it is sufficient to graph the results in the two dimensions that most closely represent the actual relatedness of individuals to each other. Figure 2 is a PCoA of the 182 Samoyed that were studied and separated by geographic origin. Dogs from North America (NA) were distributed across the plot, while dogs from Europe (EU) tended to segregate above the center of the X- axis and dogs from Australia to the right of the center of the Y axis as genetic outliers. Although there was some geographic differentiation, Samoyed across the world appear to be relatively homogeneous unlike breeds such as the Italian Greyhound [16], but like Standard Poodles [14].

#### Internal relatedness among the 182 Samoyed

**Fig. 3:**
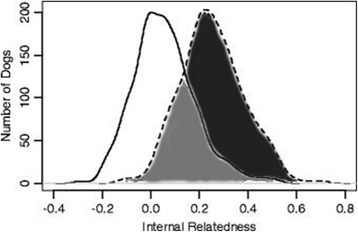
Internal relatedness (IR) scores for 182 Samoyeds from various regions of the world (solid line). The IR scores were also adjusted (dashed line) to reflect the amount of genetic diversity present in village dogs of the world that is still retained in contemporary Samoyed. The two graphs would be superimposed if Samoyed and village dogs were totally related and completely separate if there was no genetic relationship. The light-shaded area reflects the degree of genetic relatedness of Samoyed to village dogs based on allele sharing at the 33 autosomal STR loci (43%), while thee dark shaded area is a measure of non-relatedness (57%) or potential genetic diversity lost during breed evolution

Internal relatedness (IR) was also calculated from allele and allele frequencies of 182 Samoyed obtained from analysis of the 33 autosomal STRs. Internal relatedness is an indirect measurement of how related a dog’s parents were to each other. Individual IR values can then be graphed to show the average IR values for the population (Fig. 3). An IR value of −1.00 would indicate that the parents were totally unrelated at each of the 33 genomic STRI loci, while a value of +1.00 would indicate genetically identical parents. A mean value of 0.25 would be equivalent to being an offspring of brother to sister mating.

The most outbred (least related parents) individual among the 182 dogs had an IR score of −0.252 and the most inbred (most related parents) dog had an IR score of +0.502 (Table 4). The average Samoyed among the 182 dogs tested had an IR score of 0.056. The graph for IR values is reasonably symmetrical with one half of the dogs scoring lower than +0.056 and one-half scoring over +0.056. This indicates that the population contained more inbred than outbred dogs, mirroring the positive coefficient of inbreeding (F) derived from standard genetic indices (Table 3). However, the IR scores provided a more precise estimate of the degree to which individual dogs were inbred or outbred than the standard fixation indices. One quarter of Samoyed had IR scores between 0.132 and 0.502, which indicates a significant degree of parental relatedness considering that a value of +0.250 would be equivalent of offspring of full-sibling mating that happened by chance from a large random breeding population. In contrast, one quarter had IR scores between −0.042 and −0.252 and would have parents that were less related to each other than most dogs in the population.

**Table 4:** IR vs IRVD comparison for Samoyed (*n* = 182)

	IR	IRVD
Min.	−0.252	−0.12
1st	−0.042	0.185
Median	0.042	0.268
Mean	0.056	0.2743
3rd	0.132	0.355
Max.	0.5021	0.677

Internal relatedness scores can be used to approximate how much genetic diversity has been lost during a breed’s evolution by comparing their allele frequencies with the incidence of the same alleles in contemporary village (indigenous, landrace) dogs. The assumption would be that contemporary free-roaming dogs and randomly breeding village from Lebanon, Iran, Taiwan, Thailand, Philippines, Brunei, Cook Islands and Bali [27] would possess all of the genetic diversity present in the Samoyed founders. The resulting IR village dog (IRVD) values can be presented in a graphic (Fig. 3) or statistical manner (Table 4). The least related (most genetically diverse) individual among the Samoyed had an IRVD score of −0.120 and the most related (least genetically diverse) dog scored +0.677 (Table 4). The mean IRVD value at the peak of the graph is +0.268, therefore, over one-half of Samoyed had parents that were genetically comparable to full siblings from a random breeding village dog population (IRVD = +0.250). Values as high as +0.677 would only occur if the full-siblings were themselves offspring of closely related dogs. Based on graphic portrayal (Fig. 3), contemporary Samoyed possess about 43% of the diversity present in modern village dogs.

### DLA class I and II haplotypes among 182 Samoyed as determined by linked STR loci

Thirteen STR-associated DLA class I haplotypes were identified among the 182 Samoyed (Table 5). Nine of these haplotypes have been found in other pure breeds of dogs, while four haplotypes have not been identified in any other breed to date (Table 5). Two of these DLA class I haplotypes, 1011 (shared) and 1152 (unique) were found in 77% of the dogs. If haplotype 1009 is included, 89% of Samoyed share the same three haplotypes. The major 1011 haplotype identified to date by our laboratory is also found in the Standard and Miniature Poodle, Golden Retriever, and Alaskan Klee Kai.^14^

**Table 5:** DLA class I and II haplotypes found in Samoyed with unique haplotypes in bold print

Class I			Class II		
ID	Haplotype	Frequency	ID	Haplotype	Frequency
1006	387/375/293/180	0.006	2002	343/327/280	0.003
1009	382/377/277/184	0.122	2003	343/324/282	0.014
1011	376/365/281/180	0.272	2007	351/327/280	0.006
1012	388/369/289/188	0.014	2015	339/327/280	0.017
1014	375/373/287/178	0.003	2022	339/327/282	0.106
1061	380/365/281/183	0.003	2024	343/323/280	0.014
1068	380/373/287/181	0.044	2042	341/324/286	0.003
1085	376/373/277/186	0.003	2050	341/327/284	0.003
1094	386/369/289/176	0.014	2053	343/324/280	0.561
**1152**	390/373/281/180	0.494	**2095**	355/322/280	0.158
**1153**	389/373/287/183	0.019	**2096**	351/322/280	0.114
**1157**	386/373/281/180	0.003	**2099**	345/324/276	0.003
**1158**	390/371/281/180	0.003			

Twelve class II haplotypes were identified in Samoyed and three of these have not yet been identified in any other breed (Table 5). The dominant DLA class II haplotype 2053 was found in 56% of Samoyed and in the Miniature Poodle, Havanese, Biewer, Golden Retriever, and Flat Coated Retriever.^14^ Seventy-two percent of Samoyed possess the 2053 (shared) or 2095 (unique) DLA class II haplotypes.

The number of class I and II haplotypes is about average for pure breeds that have been studied to date by our group.^14^ The total number of different DLA class I and II haplotypes differed among North American, European and Australian Samoyed as a reflection of population size. However, the most common haplotypes were shared in all three populations, while minor haplotypes were missing in in the smaller populations (data not shown).

Although it appeared that Samoyed were being purposely selected for certain DLA class I and haplotypes, the comparative incidence of haplotypes does not reflect how randomly these specific haplotypes were segregating in the population. Therefore, a standard genetic assessment was made of the allele and allele frequencies of the seven loci associated with the DLA class I and II regions (Table 6). The Na for the seven loci was 6.14 and the Ne was 2.09. The low Ne reflected the high incidence of a small number of haplotypes; however, the Ho was 0.462 and the He 0.485, with the inbreeding coefficient F of +0.045. Although there was a marked imbalance in the incidence of DLA class I and II haplotypes, the F value for the seven DLA class I and II STR loci was identical to the F value for the 33 genomic STR markers. These findings again support the conclusion that the majority of the 182 Samoyed were products of individuals that were as unrelated as possible given the limited genetic diversity, balanced by a small number of more inbred and more outbred individuals.

**Table 6:** F-statistics for Samoyed (*n* = 180–182) using 7 STRs in the DLA class I and II regions

Locus	N	Na	Ne	Ho	He	F
DLA I-3CCA	182	9.000	2.946	0.626	0.661	0.052
DLA I-4ACA	180	6.000	2.424	0.556	0.588	0.054
DLA I-4BCT	182	5.000	1.620	0.385	0.383	−0.005
DLA1131	182	8.000	1.618	0.379	0.382	0.007
5ACA	182	6.000	2.448	0.560	0.591	0.052
5ACT	182	4.000	2.312	0.516	0.568	0.090
5BCA	181	5.000	1.288	0.210	0.224	0.062
Mean	181	6.143	2.094	0.462	0.485	0.045
SE		0.670	0.224	0.055	0.060	0.012

### Studies of ARAI in Samoyed

#### Clinical characterization of disorder

Fourteen affected dogs, 11 from the USA and 3 from Europe, were identified for this study. The disease was characterized by one of the authors (BS), and with pictures provided by some breeders and owners.

**Fig. 4:**
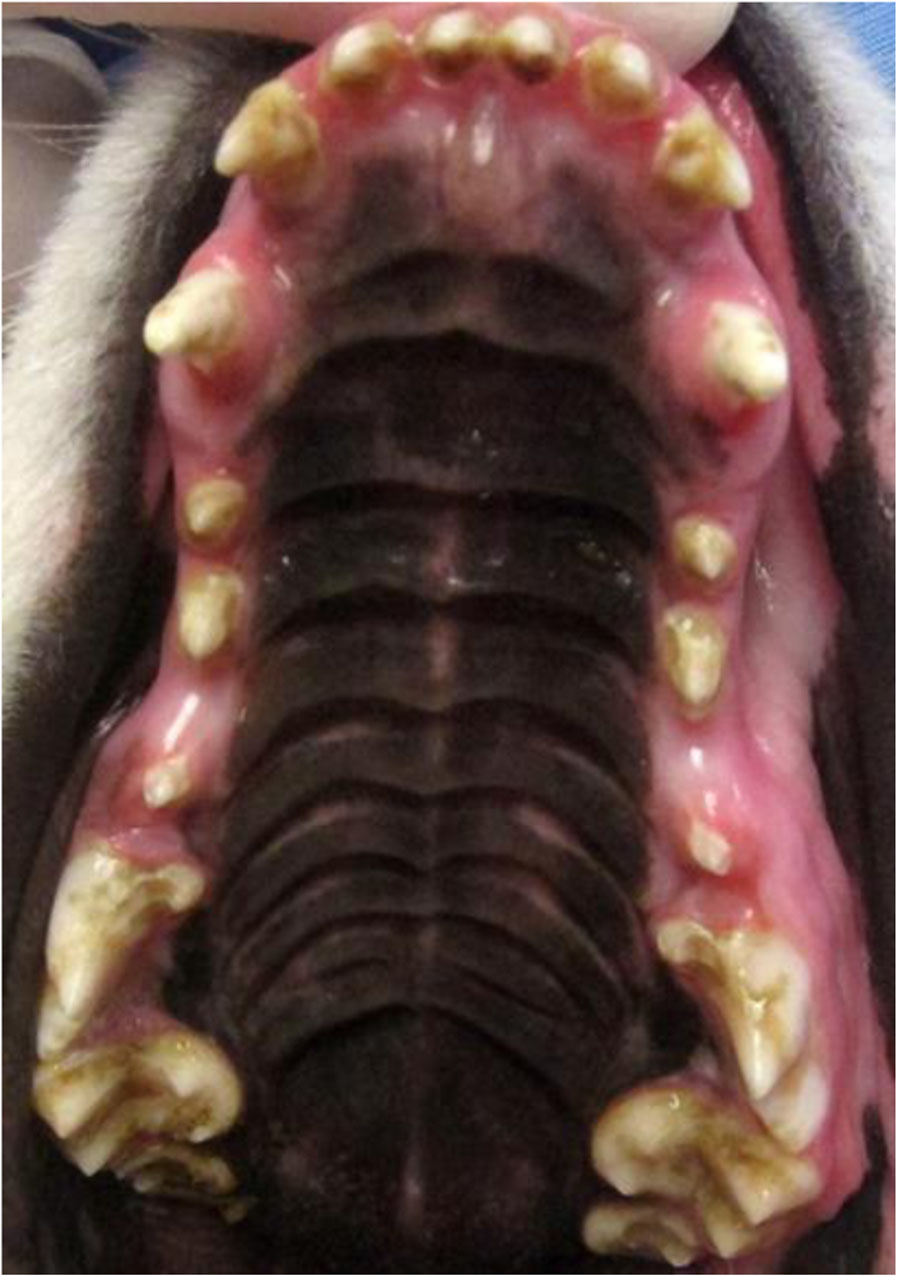
Samoyed with typical lesions of heritable enamel hypoplasia. The teeth are noticeably discolored; smaller, blunted, and further apart; tooth surfaces are irregular. This dog has received regular dental care and therefore dental tarter is largely absent and the gums remain in good health

**Fig. 5:**
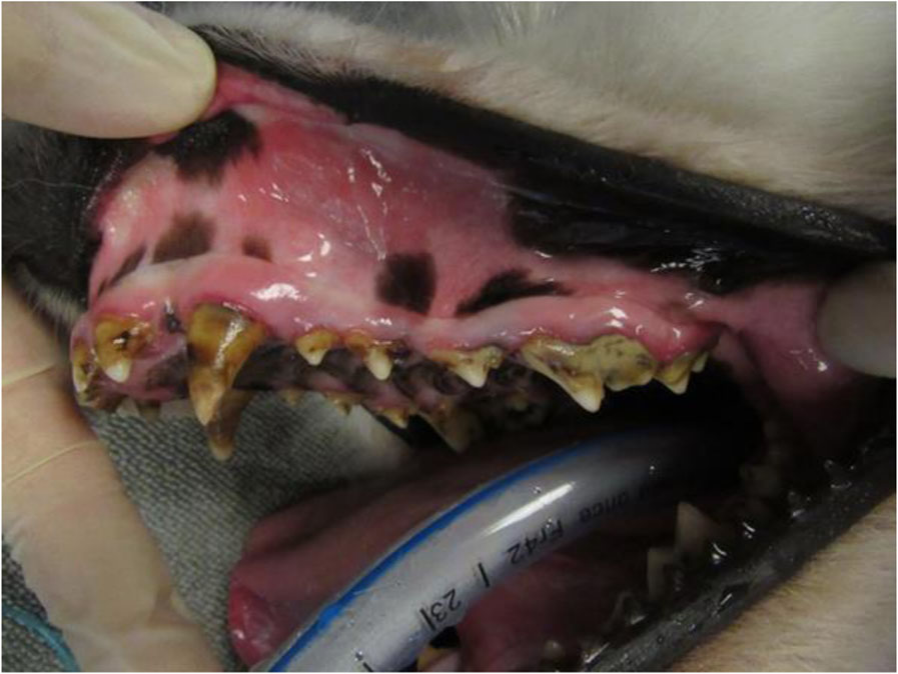
Samoyed with enamel hypoplasia and severe discoloration of the teeth, excessive tarter accumulation, and swollen gums due to gingival disease

The deciduous teeth in Samoyed enamel hypoplasia are normal, but abnormalities are apparent in adult teeth immediately upon eruption. An early sign of the disorder is bad breath. Figure 4 shows the typical appearance of the teeth of a Samoyed with heritable enamel hypoplasia in a dog that has received regular dental care. The teeth are discolored and the surfaces pitted in places where enamel is either missing or thin. The teeth are often blunted in appearance and the spaces between teeth are increased due to thinning of the enamel layer. Tarter and calculus tend to build up rapidly in the irregular tooth surfaces in the absence of routine dental care (Fig. 5). Gingivitis and swelling of the gums is a common accompanying problem (Fig. 5) and often progresses to more advanced periodontal disease. Dental caries can occur and may extend into the dental pulp. Tooth loss can occur due to severe abrasions, fractures and accompanying tooth root infections.

#### Genome wide association study and identification of causative mutation in SCL24A4

**Fig. 6:**
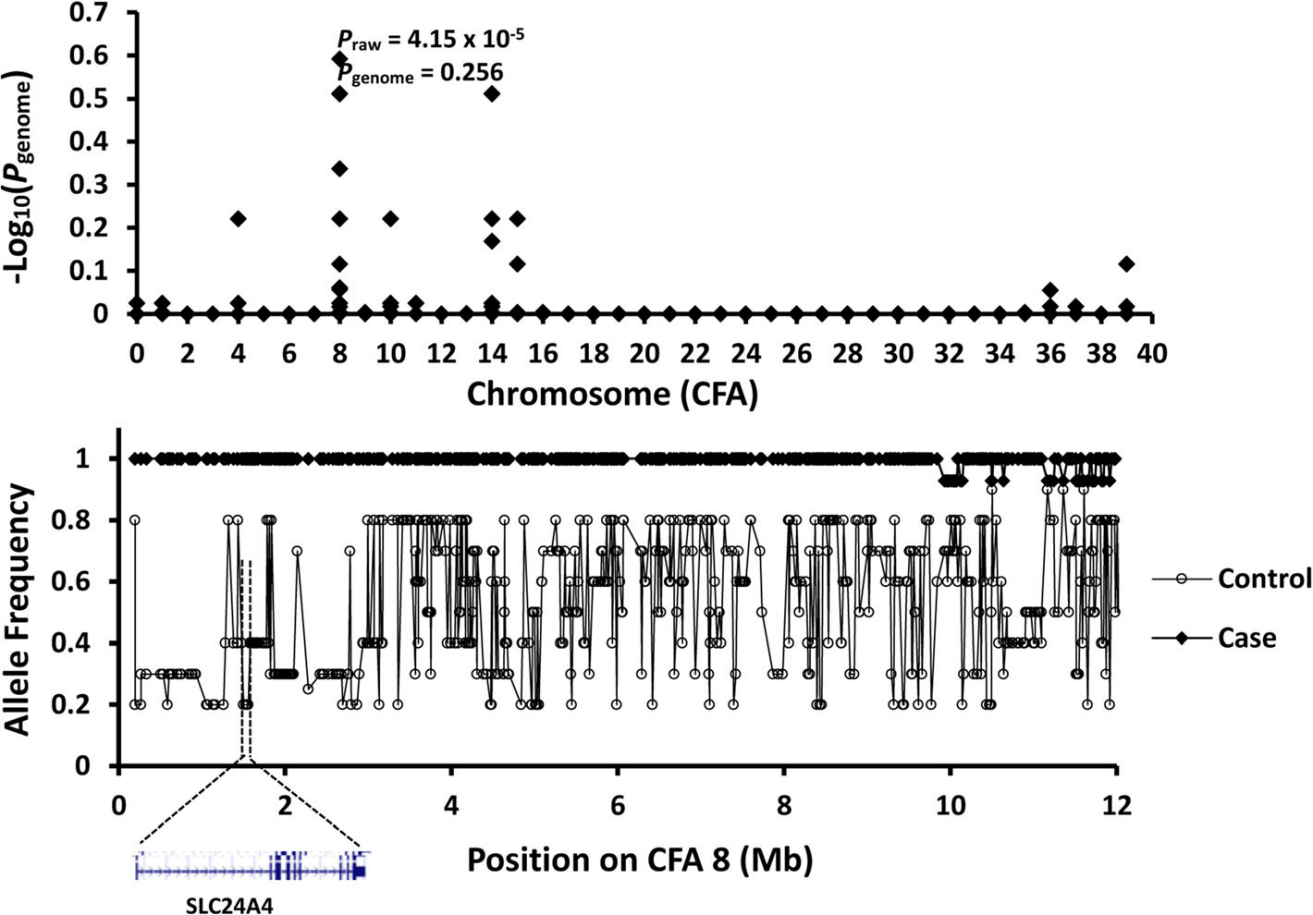
(Upper) Manhattan plot of GWAS using seven Samoyed with enamel hypoplasia five unrelated healthy controls showing a genome-wide association on CFA 8. (Lower) Allele frequency in the 12-Mb region of CFA 8 for enamel hypoplasia (solid diamond) and control (open circle) and the location of a known candidate *SLC24A4* for a form of autosomal recessive amelogenesis imperfecta in humans

A GWAS was conducted on seven Samoyed with abnormal teeth compatible with enamel hypoplasia and five dogs with healthy teeth. The GWAS showed a peak made up of a string of SNPs in a 12 Mb region of chromosome 8 with a Praw value of 4.15 × 10^−5^, however the adjusted association was below significance (Pgenome = 0.256) (Fig. 6 upper). A graph of allele frequency for the 12 Mb region of CFA8 showed an extended region of homozygosity (selective sweep) in a gene (*SCL24A4*) known to cause enamel hypoplasia in humans (Fig. 6 lower). Primers were designed for amplification and sequencing the coding sequences of canine SLC24A4 (Table 1).

**Fig. 7:**
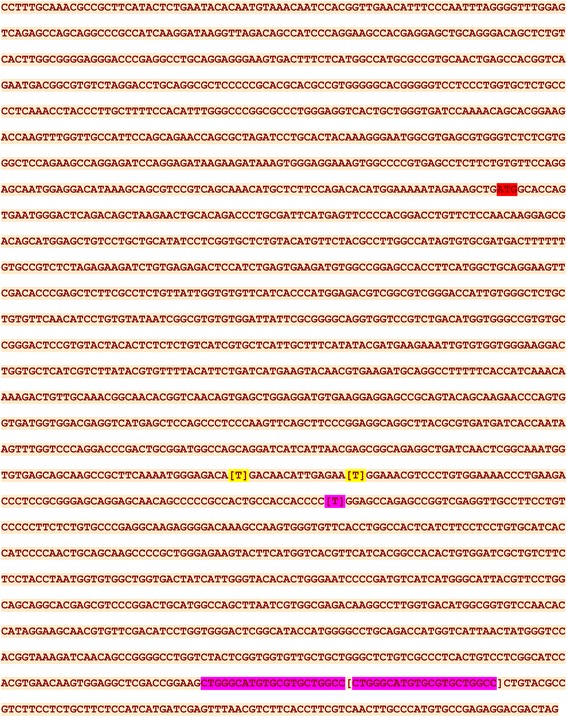
Coding sequence of *SCL24A4* from a dog suffering from enamel hypoplasia. There are two synonymous nucleotide changes (indicated in parentheses), an asynonymous change (C to T) in exon 12 changing amino acid from proline to leucine, and a 21 bp insertion in exon 17

**Fig. 8:**
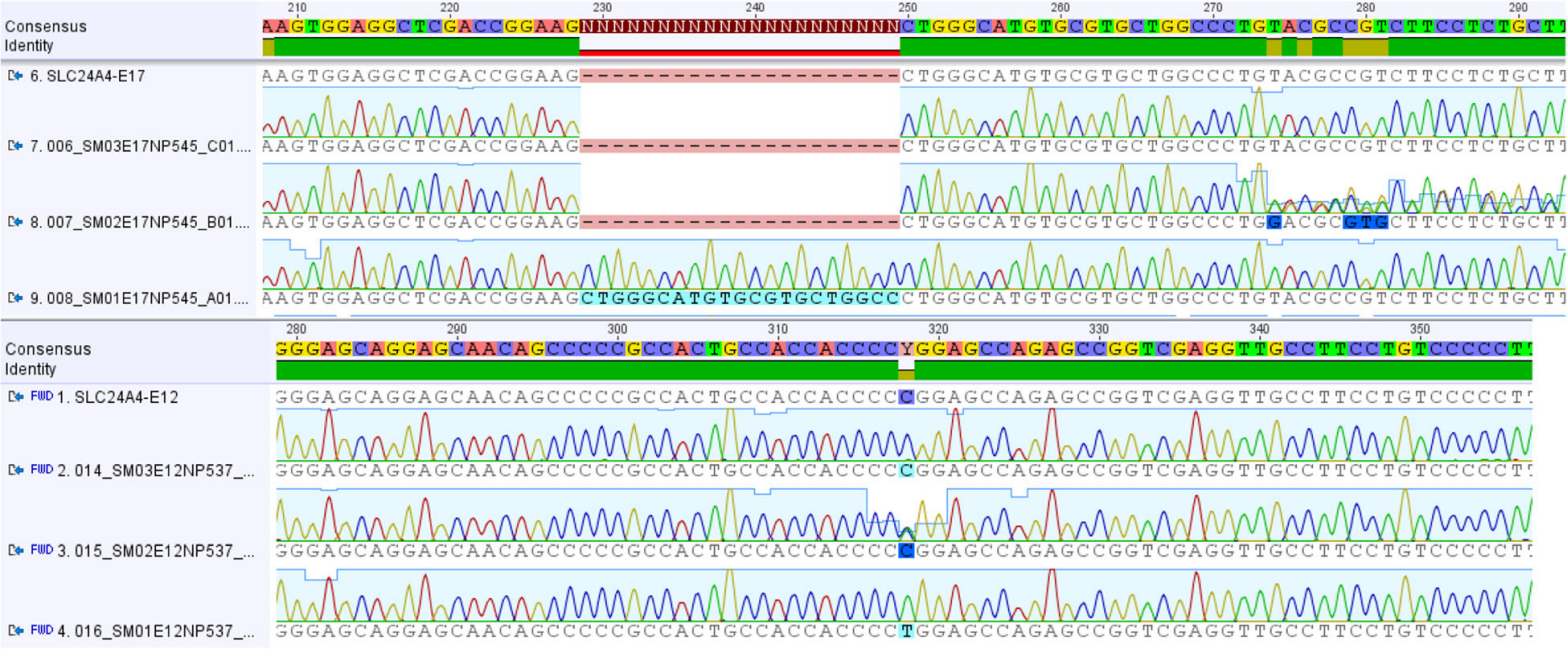
SLC24A4 chromatograms of ARAI affected (SM01) and its healthy sibling (SM03) and his Dam (SM02). There is a 21 bp duplication in exon 17 of SM01 that is not present his healthy sibling. SM01 also has a C to T SNP causing amino acid change in exon 12 that does not occur in his sibling. The Dam is heterozygous for both sites

The PCR products were then sequenced and two synonymous nucleotide changes (indicated in parentheses), an asynonymous change (C to T) in exon 12 changing amino acid in exon 12 from proline to leucine, and a 21 bp insertion in exon 17 were identified (Fig. 7). Chromatograms of an enamel hypoplasia affected dog (SM01), its healthy sibling (SM03) and Dam (SM02), for the 21-nucleotide insertion in Exon 17 are presented in Fig. 8.

### Incidence of *SCL24A4* mutation in Samoyed

A test was developed for detecting the 21 bp duplication in exon 17 of *SCL24A4* using capillary gel electrophoresis that would allow it to be incorporated into the same panels used for determining alleles at the 33 autosomal and seven DLA class I and II associated STR loci and amelogenin. The test could accurately detect dogs that did not have the mutation and dogs that were heterozygous or homozygous for the mutation. The 14/182 (7.8%) dogs that were presumed to have enamel hypoplasia based on physical examination and DNA testing all were homozygous for the mutation. Twenty of 168 (12%) heathy dogs were found to be heterologous for the mutation and most were parents or known close relatives of affected dogs.

### Genetic relationship of affected dogs by PCoA

**Fig. 9:**
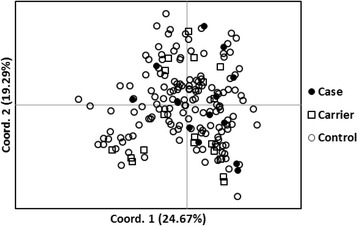
PCoA plot of 148 normal, 11 ARAI affected, and 20 ARAI carriers based on data from the 33 genomic STRs

Enamel hypoplasia affected and carrier dogs were compared to the healthy Samoyed by PCoA (Fig. 9). The analysis showed that affected and carrier dogs were distributed randomly across the entire population. This was another indication that the defect had been in the population for some time - the oldest affected dog was 11 years of age.

### Effect of eliminating affected and carrier dogs on genetic diversity in the total population

Breeders have three options when faced with a deleterious autosomal recessive mutation: 1) do nothing, 2) eliminate the mutation from the entire breeding population through testing, and 3) avoid producing homozygous puppies by testing and breeding affected and carrier dogs only to normal dogs. The first option only applies to a large and genetically diverse population with a very low incidence of carriers that is under strict random breeding, thus keeping the number of affected dogs low. Option two is preferred when a test for the mutation is available, the carrier incidence is not high, and there is sufficient genetic diversity to avoid losing diversity in the process. The third option is preferable when genetic diversity is low, the population size is small, the incidence of the mutation is high, and the loss of diversity would be great. The best approach can be easily determined by measuring changes in basic genetic parameters when the affected and carriers are removed from the computation. Table 7 shows the effect on genetic diversity among 168 healthy Samoyed before and after the 20 carriers were removed, and assuming a carrier incidence of 12%. There was no significant change in the average number of alleles per locus (Na), the effective alleles (Ne) per locus, observed and expected heterozygosity (Ho and He) or in the inbreeding coefficient F. Therefore, if the actual carrier incidence was 12% or less in the population, the mutation could be safely eliminated without affecting existing genetic diversity. Table 8 shows the same type of calculation, but for DLA class I and II frequencies. The haplotype frequencies were also unchanged after the carrier dogs were eliminated.

**Table 7:** Genetic assessment of removing carrier dogs from a healthy appearing population of Samoyed if no more than 12% of the dogs are carriers. Mean ± one standard error

Pop		N	Na	Ne	Ho	He	F
Normal	Mean	148	5.939	3.230	0.612	0.638	0.038
	SE		0.351	0.212	0.027	0.028	0.011
Normal + carriers	Mean	168	5.970	3.240	0.616	0.640	0.038
	SE		0.349	0.211	0.027	0.028	0.011

**Table 8:** Change in relative DLA class I and II haplotype frequencies before and after removing the 12% of dogs that are carriers of the enamel hypoplasia mutation

DLA Class I			DLA Class II		
Haplotype	Normal	Normal + Carrier	Haplotype	Normal	Normal + Carrier
1006	0.007	0.006	2002	0.003	0.003
1009	0.130	0.127	2003	0.017	0.015
1011	0.223	0.259	2007	0.007	0.006
1012	0.017	0.015	2015	0.021	0.018
1014	0.003	0.003	2022	0.110	0.108
1061	0.003	0.003	2024	0.007	0.015
1068	0.045	0.045	2042	0.003	0.003
1085	0.003	0.003	2050	0.003	0.003
1094	0.007	0.015	2053	0.603	0.566
1152	0.541	0.506	2095	0.140	0.151
1153	0.014	0.012	2096	0.082	0.108
1157	0.003	0.003	2099	0.003	0.003
1158	0.003	0.003			

## Discussion

### Genetic diversity in contemporary Samoyed

The objective of this study was to characterize an autosomal recessive mutation that appeared in Samoyed and to determine how genetic diversity and inbreeding may have contributed to its origin, spread, and potential effect on existing diversity. Therefore, genetic diversity in contemporary Samoyed was first evaluated. Genetic diversity can be measured from pedigrees, but if mating is non-random, their accuracy and quantity must be high [28]. Pedigrees used for genetic diversity must also include all founders that contributed to the breed [29]. Therefore, it is increasingly common to include both deep pedigree and DNA analysis in studies involving genetic diversity. Extensive pedigrees and large SNP arrays have been used to determine genetic diversity and genome substructure in dog breeds such as the Bull Mastiff [30]. One problem with large SNP arrays is to find ways to present complex data sets in a simple manner [31]. Data from STRs (microsatellites, single sequence repeats) is easily understood and has been used in combination with pedigrees in genetic analyses of breeds such as Standard Poodles [14] and Italian Greyhound [16]. STRs are also considered still informative in plant genetics [32] and research on certain meat breeds of cattle found STRs to be equivalent to pedigrees [33]. STRs were used in-lieu of pedigrees in genetic studies of the Bulldog [13] and several other breeds.^14^ STRs are also economical and can work with limited amounts of DNA and even poor quality DNA. Unlike STRs, SNPs cannot interrogate highly polymorphic regions such as the DLA. Therefore, genetic diversity in the present study was determined with small panels of 33 STR loci on 25/38 autosomes and 7 STRs defining the DLA class I and II regions on CFA12.

It is uncertain how many dogs constituted the founding population for the Samoyed, although an analysis of pedigrees from dogs registered in the USA from 1990 to 1999 by Bell (2002)^18^ showed an increasing number of the same individuals in earlier generations. This suggested a small founder population. However, an analysis of a large number of pedigrees as part of this same study showed that the average inbreeding coefficient of contemporary Samoyed was 9.94% +/− 7.64 and it was concluded that “that Samoyed has acceptable overall breed-wide diversity.”^18^ Sorsa,^19^ using SNP arrays (MyDogDNA pilot analysis), found that the genetic diversity of Samoyed (also known as Bjelkier in Europe) was above the median of 3000 dogs of all breeds in their database and concluded that “even though the population size of less than 50 dogs (used in the study) is very small, it gives an initial indication that the active Samoyed breeders and owners would have succeeded in their systematic efforts for preserving diversity.”

The most extensive population and genetic study of the Samoyed was reported by the UK kennel club for the period between 1980 and 2014.^10^ There were about 200 Samoyed registered in the UK in 1980, but this rapidly increased to a peak of over 1200 dogs by 1995. The breed appeared to lose popularity after this time and only about 300 Samoyed were registered in 2014. The population increase was closely linked to the number of puppies produced each year by a single sire.^10^ A single sire averaged 2.32 puppies in 1980, 10.56 offspring in 1994 and that dropped back to 7.05 in 2014. An observed and expected inbreeding coefficient was calculated and graphed for the period 1980–2014.^10^ The inbreeding coefficient is the probability that two alleles at a given gene locus in random members of a population are identical. In 1980, the observed inbreeding coefficient was 0.022, while the expected inbreeding coefficient was around 0.015.^10^ The difference was 0.007, or a 0.7% rise in identical alleles in the population prior to the population bubble starting in 1980. In contrast, the observed inbreeding coefficient at the peak of the population in 2001 was 0.11 and the expected inbreeding coefficient to 0.06, a calculated difference of 0.05. Therefore, allele sharing based on these figures increased by 4.3% (0.05–0.007) from 1989 to 2001, while the observed coefficient of inbreeding dropped to 0.08 in 2014 as the population numbers declined. Using the figures of this study,^10^ the expected coefficient of inbreeding at 2014 was around 0.06, a difference of 0.02, indicating a trend towards more random breeding. Overall, allele sharing among UK Samoyed increased from 0.7% in 1980 to 5% in 2001 and back to 0.2% in 2014. The author of UK kennel club survey concluded*: “As with most breeds, the rate of inbreeding was at its highest in this breed in the 1980s and 1990s. This represents a ‘genetic bottleneck’, with genetic variation lost from the population. However, since 2000 the rate of inbreeding has slowed and even declined slightly, implying maintenance and even some replenishment of genetic diversity (possibly through the use of imported animals).”* ^10^ There are two problems with this conclusion. Inbreeding and genetic variation (diversity) are different things. It is possible to expand a portion of the population by rapid inbreeding without losing any original genetic diversity. Inbreeding can be reversed if even remnants of original diversity survive, but genetic diversity that is permanently lost through inbreeding can never be replaced from within the population. The conclusion of the UK Kennel Club study was also misleading because it implies that allele sharing at each locus is not a problem in the breed. Based on DNA testing, the average percentage of homozygous alleles across the 33 autosomal STR loci in contemporary Samoyed was 39% (SD = 9.5%), while the average percentage of homozygous alleles was 54% (SD = 38%) for the 7 STR loci in the DLA class I and II regions. Therefore, allele sharing in Samoyed is greater than the percentages indicated by this 1980–2014 population study by the UK Kennel Club. Given a high level of pre-existing allele sharing, the loss of even small amounts of heterozygosity in such population bubbles can have a dramatic effect. Such bouts of inbreeding are also times when new mutations occur and/or when existing deleterious mutations are amplified to clinical levels. It is possible, given the timelines that the causative mutation for ARAI in Samoyed occurred during this population bubble.

The increased inbreeding observed during the 1980s and 1990s is typical of what occurs when a breed becomes popular and the need for puppies and their value greatly increases. Professional breeders usually blame commercial breeders for the great increase in inbred dogs during such periods, and there is a measure of truth to this belief. This is exemplified by what happens when popularity declines. Commercial breeders leave the market and professional breeders tend to return to more random breeding practices.

The same UK Kennel club survey also provided an estimate of the effective population size for Samoyed of 64.7 dogs.^10^ Effective population size, founder equivalents and founder genome equivalents are related terms that usually refer to the “number of equally contributing (randomly breeding) founders that would be expected to produce (maintain) the same genetic diversity as in the population under study [29].” The question is whether 64.7 Samoyed, possessing the present spectrum of genetic diversity and freely breeding, would be sufficient to maintain the current level of genetic diversity. A minimum number comes from the 50/500 rule proposed by Franklin in 1980 [34]. The “50” part of the 50/500 rule states that inbred populations with an effective population size under 50 are at immediate risk of extinction if the population size should fall below 500 individuals. Breeding within such a small inbred population, whether it is random or nonrandom, can quickly force a small population into a downward spiral of lost diversity (i.e., an extinction vortex). In purebred dog terms, a genetic bottleneck such as a popular sire effect can lead to a decrease in effective population size, while a catastrophic event such as a world war or loss of popularity can reduce the population size to fewer than 500 dogs. The 50/500 rule has more application to wild populations subject to survival of the fittest, while most dog breeds can be sustained by considerable human intervention and sophisticated veterinary care, an example being the Bulldog [13]. Nonetheless, the world-wide number of Samoyed is well over 500 dogs and is presumably sufficient to maintain existing genetic diversity if properly managed to avoid further cycles of intense inbreeding.

The present studies confirm and expand upon the findings of others regarding genetic diversity in the Samoyed. One hundred eighty-two dogs from different geographic regions of the world should provide a reliable measure, based on other breeds studied by our group, of the incidence of all major and most minor alleles present at each of the 33 autosomal STR loci and comparative incidence of DLA class I and II haplotypes.^14^ The amount of genetic diversity found in contemporary Samoyed was greater than our group found in breeds such as Flat-coated retrievers, Doberman, and Bulldog; similar to the Alaskan Klee Kai, Biewer, and Black Russian Terrier; and less than Standard and Miniature Poodle, Havanese, Akita, Golden Retriever, and Italian Greyhound.^14^ There was evidence for some geographic differentiation of European and Australian dogs but the numbers, especially of Australian dogs were too small to confirm this finding. However, this differentiation was only slight and far less identifiable than between Japanese and American Akita as tested by our laboratory^20^ or American and European Italian Greyhound [16]. The geographic differentiation was more like that demonstrated for English and American Standard Poodle [15]. The genetic homogeneity of Samoyed from disparate parts of the world was somewhat surprising given the fact that the breed started in several different countries at about the same time. This suggests that there has been a great deal of international exchange of Samoyed over the last century.

Allele frequencies at each of the 33 autosomal STR loci indicated that 70–90% of the population was closely related to each other. Without accurate information on breed founders, it is not possible to say whether there were few founders at the start or that many founders were lost as the breed evolved over the last century. The DLA haplotypes confirmed that the contemporary Samoyed has been heavily selected for two lineages. The DLA region is in strong linkage disequilibrium and each class I and II haplotype is inherited largely unchanged over a long period of time from sire and dam. About 90% of all Samoyed tested shared three class I and four class II haplotypes. One class I (1152) and one class II (2053) haplotypes were found in over 50% of the dogs. The major 1152 haplotype along with three less common haplotypes were unique to the breed among those identified to date by our group.^14^ The major 2053 haplotype was also found in other breeds, while the Samoyed possess three unique class II haplotypes. These unique and/or major haplotypes are most certain to belong to founder dogs that had features strongly engrained in the breed standard and have been diligently maintained. A standard genetic assessment of the 7 STR loci associated with these haplotypes indicates that these haplotypes, although highly unbalanced in proportion, are largely in breed-wide equilibrium.

### Incidence of mutation responsible for ARAI

It is uncertain when the first case of enamel hypoplasia occurred in the breed, although some recall possible cases as far back as 1985. Based on the oldest age of affected dogs, the causative mutation has been present for several generations; the oldest affected dog was 11 years of age and assuming the parents were 4–5 years old at its birth. Both parents also had to be either heterozygous or homozygous for the mutation, pushing the timeline back several more generations. Although the mutation may be old, the occurrence of diseased dogs has been apparently increasing of late. This suggests that the mutation has been under recent positive selection, most likely in association with a desired trait. There are rumors that an affected popular sire may be involved, but this was not pursued in the present study. However, this is where pedigrees associated with DNA testing could be highly informative as to the precise origin of the mutation and the cause for its recent spread. Detective work of this type was used to trace the origins of sebaceous adenitis and Addison’s disease in the Standard Poodle [14]. The exact incidence of affected dogs is also not known. The incidence of carriers among the healthy dogs that were tested was around 12%, which would make the incidence of affected dogs for an autosomal recessive disease 0.12 × 0.12 × 0.25 = 0.0036 or 3.6/1000. If this figure is correct, the incidence of affected dogs is below the incidence of 1–2% or greater when deleterious autosomal recessive traits become of concern in other breeds.

### Genetic basis for ARAI in Samoyed

Enamel formation is a complex process involving many genes working in unison and at various stages of tooth development. Wright and colleagues [35] listed 91 conditions in *Online Mendelian Inheritance in Ma*n as having an enamel phenotype, and of those, 71 have a known molecular etiology or linked genetic loci. The complexity of enamel formation allows for mutations in many different genes to cause a similar defect. Therefore, it is not surprising that enamel hypoplasia has been observed in many breeds, although only one other mutation causing a heritable enamel hypoplasia has been characterized to date, an ARAI involving the enamelin gene (*ENAM*) in Italian Greyhounds [17]. One reason for this has been the relatively recent growth of the specialty of veterinary dentistry and the previous tendency to dismiss such cases as due to non-heritable causes such as canine distemper, high fevers, drugs or traumas occurring during puppy-hood [36]. The enamel dysplasia described up to this time has also been relatively mild and easily confused with plaque build-up and tooth discoloration in dogs. The enamel hypoplasia in Italian Greyhound is much more common, but also less severe [17]. An even milder enamel hypoplasia that appears to be heritable has been reported in the Standard Poodle [37]. The complexity of genes involved in enamel formation, and the large possible numbers of mutations affecting enamel is reminiscent of the large number of genes associated with vision and the many genetic causes of progressive retinal atrophy [38].

Two potential deleterious mutations in *SCL24A* on CFA8 and in strong linkage disequilibrium were ultimately identified in ARAI affected dogs, an asynonymous change (C to T) in exon 12 changing amino acid proline to leucine and a 21 bp duplication in exon 17. The leucine to proline change in exon 12 has been reported in GenBank in golden snub-nosed monkey (XP_010382802, XP_010382799, XP_010382800) and camel (XP_006184249, XP_006184251) and was assumed to be a polymorphism present in normal animals and not deleterious. The insertion in exon 17 that caused a 21 bp insertion was in the terminal transmembrane region of *SLC24A4* and would be more likely to inhibit function of the 4SCL24A4 protein, which transports one Ca++ and K+ ion in exchange for four Na + ions [41]. A duplication of this size in terminal exon 17 would presumably change the size of an internal-membrane portion of the protein.

The large run of homozygosity (selective sweep) containing these *SCL24A4* mutations was highly conserved among all the affected Samoyed tested, indicating that the region possesses many genes and gene polymorphisms that define the breed phenotype in some manner. It is also likely that a polymorphism that was deemed favorable to the breed, most likely involving a conformational trait, also occurred in or near this same region and that it was amplified as well. The number of these selective sweeps varies greatly in number and size between breeds. However, sweeps become larger and more numerous as the phenotype deviates more and more from the ancestral dog. As such, the Bulldog and Bull Mastiff have numerous large selective sweeps [13, 39], whiles the Standard Poodle, with the same level of SNP homozygosity, has fewer and smaller selective sweeps in its genome [13, 40]. The presence of numerous selective sweeps, when coupled with frequent changes in desired show traits and artificial positive selection for those phenotypes, are significant factors behind the high incidence of genetic disorders in pure breeds of animals. Dog breeds have undergone and continue to undergo the most phenotypic change, also have among the highest incidence of heritable disease [13]. If the Samoyed should continue to lose genetic diversity and be subjected to continued bouts of conformational change, other simple genetic diseases will occur. As a dog breed becomes more inbred the incidence of complex genetic traits will also rise [13, 14, 16].

### Comparison of Samoyed ARAI with SLC24A4 ARAI in people

**Fig. 10:**
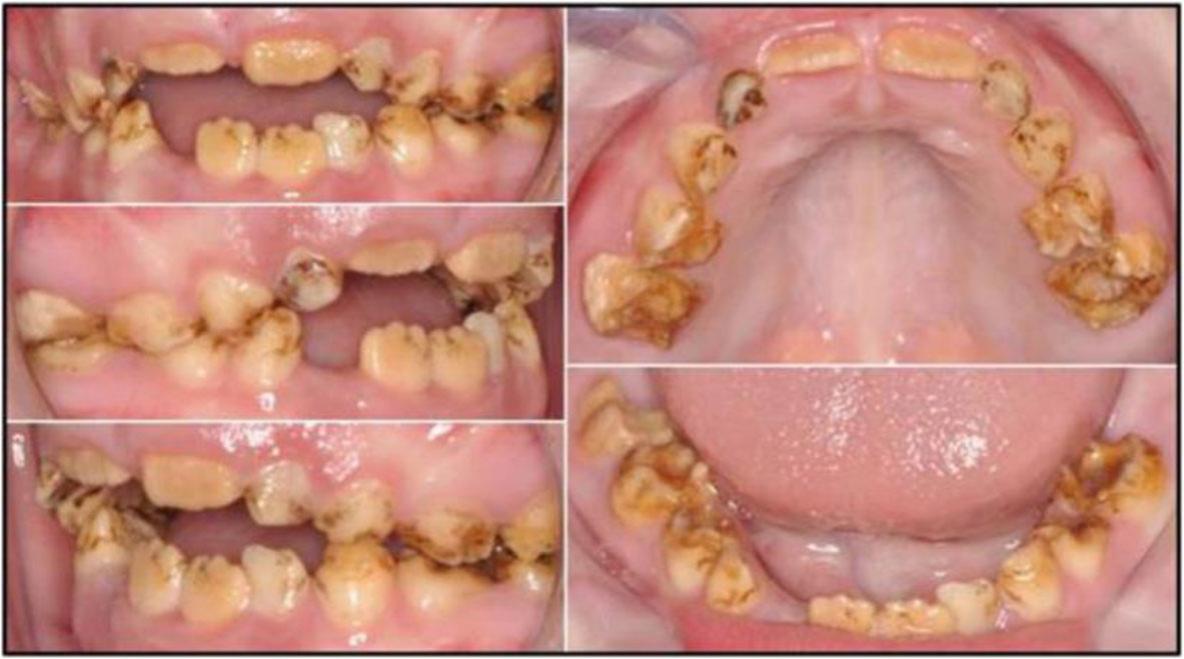
ARAI in a six-year-old girl caused by a missense mutation in *SLC24A4*. Reprinted with permission from: Herzog CR et al. Hypomaturation amelogenesis imperfecta caused by a novel *SLC24A4* mutation. Photographs from: Oral Surg Oral Med Oral Pathol Oral Radiol. 2015 Feb;119(2):e77-e81

*SLC24A4* has only been recently identified as a cause of amelogenesis imperfecta (AI) in humans. Parry and colleagues [41] identified a missense mutation in the ion binding site of SLC24A4 in a family with hypocalcified teeth. Seyman et al. [42] described a 10 kb deletion in covering exons 15, 16 and most of 17 in a family with brown discoloration of the teeth. A third mutation was also identified as a missense T > G mutation in *SLC24A4* causing a leucine to arginine switch at position 436 and closely resembles the clinical appearance of the enamel hypoplasia in Samoyed [43] (Fig. 10).

### Strategy for control of ARAI in Samoyed

What should breeders do when a genetic mutation such as this occurs in their breed? The answer depends on how much genetic diversity will be lost in the attempt to eliminate it from the breed. In the case of Pug Dog Encephalitis, the recessive heritable associations to risk for the disease were present in one third of the dogs and the breed was limited in diversity [44]. Therefore, a decision was made to breed away from homozygotes, while maintaining the trait in a heterozygous state. The outcome of eliminating the SCL24A allele was tested in the present study by creating test populations containing a known proportion of carrier dogs, and then measuring genetic diversity before and after the carrier population is removed. The results of such testing indicated that Samoyed breeders could easily eliminate the trait without loss of genetic diversity due to the low incidence of the mutation at present time.

## Conclusions

Based upon the analysis of autosomal and DLA-related STRs, Samoyed have a lower level of genetic diversity than estimated from prior pedigree or SNP-based studies. Eighty percent of the 182 dogs tested shared two alleles at each of 33 autosomal loci and three to four DLA class I and II haplotypes. This lack of genetic diversity, when coupled with bouts of human-directed artificial selection for favorable phenotypic traits, may have encouraged the appearance of a deleterious genetic disorder. A novel form of heritable enamel hypoplasia has been detected with increasing frequency in Samoyed and the causative autosomal recessive mutation has been characterized. The mutation appears to be many generations old, but recently amplified by positive selection for a linked desired trait. A genetic test has been developed for identifying the carriers, which will enable the breeders to eliminate the disorder by selective breeding.

## Abbreviations

AKC: American Kennel Club
ARAI: Autosomal recessive amelogenesis imperfecta
DLA: Dog Leukocyte antigen
F: Inbreeding coefficient
IR: Internal relatedness
IRVD: IR adjusted for diversity lost since village dog origin
*SCL24A4*: Solute carrier 24
STR: Short tandem repeat
UKC: United Kennel Club
VGL: Veterinary Genetics Laboratory, UC Davis

## Footnotes

1 Wikipedia. Samoyed (dog). At https://en.wikipedia.org/wiki/Samoyed_(dog). Accessed 04 April 2017

2 Gabb, H. The myths and the mystique surrounding early Samoyeds and their owners. At http://www.thesamoyedclub.org.nz/i6/i6-5r.htm. Accessed 04 April 2017.

3 The Samoyed Club of America. At https://www.samoyedclubofamerica.org/the-samoyed/in-depth/breed-origin-and-history/. Accessed 04 April 2017.

4 American Kennel Club. Meet the Samoyed. At http://www.akc.org/dog-breeds/samoyed/detail/. Accessed 04 April 2017.

5 American Kennel Club, meet the SAMOYED. At: http://www.akc.org/dog-breeds/samoyed/detail/. Accessed 04 April 2017.

6 What is the Samoyed? At https://retrieverman.net/2013/04/13/what-is-a-samoyed/. Accessed 20 March 2017.

7 The Samoyed Hero of the Arctic. At http://cooldogbreeds.blogspot.com/search?q=samoyed. Accessed 22 Sept 2017.

8 Images of Robert Peary expeditions to Greenland. At http://www.bing.com/images/search?q=pictures+of+peary%19s+expedition+to+greeland&qpvt=pictures+of+Peary%27s+expedition+to+greenland. Accessed 3 Apr 2017.

9 The Samoyed. At http://www.samoyed.org/sambook.html?samoyed + history. Accessed 04 March 2017.

10 The Kennel Club. *Making a difference for dogs*. Population analysis of the Samoyed breed. September, 2015. At https://www.thekennelclub.org.uk/media/686468/samoyed.pdf. Accessed 03 March 2017. And Lewis TW, Abhayaratne BM, Blott SC Trends in genetic diversity for all Kennel Club registered pedigree dog breeds. Canine Genetics and Epidemiology 2015; 2:13.

11 University of Prince Edward Island, Canine Inherited Disease Database (CIDD), Samoyed: At http://discoveryspace.upei.ca/cidd/breed/samoyed. Accessed 04 April 2017.

12 Dog breed health. Samoyed. At http://www.dogbreedhealth.com/samoyed. Accessed 20 Sept 2017.

13 2005 ISAG canine panel for parentage verification. At http://www.isag.us/Docs/consignmentforms/2005ISAGPanelDOG.pdf. Accessed 04 April 2017.

14 Veterinary Genetics Laboratory, UC Davis. At https://www.vgl.ucdavis.edu/services/dog/CanineGeneticDiversity.php. Accessed 04 April 2017.

15 Dogset: pre-designed primer sets for fine-resolution mapping and DNA sequence interrogation in the dog. At https://www.vgl.ucdavis.edu/dogset/. Accessed 04 April 2017.

16
*e!Ensembl.* At http://www.ensembl.org/index.html?redirect = no. Accessed 05 April 2017.

17 Net Primer. Premier Biosoft Intl., Palo Alto, CA, USA. At http://www.premierbiosoft.com/netprimer/index.html. Accessed 05 April 2017.

18 Bell JS. Practical Genetics for Samoyed Breeders and Owners Health Seminar presented to the Samoyed Club of America, October 15, 2002. At http://www.samoyedhealthfoundation.org/aboutus/dr-bell-s-lecture.pdf/?searchterm = bell%20lecture. Accessed 05 April 2017.

19 Sorsa S. 2013. English, Samoyed, Bjelkier, genetic diversity, genetic lineage, DNA analysis. At http://www.mydogdna.com/blog/exploring-genetic-diversity-samoyeds-genome-wide-dna-analysis. Accessed 09 April 2017.

20 Genetic diversity testing for Akitas. At https://www.vgl.ucdavis.edu/services/dog/GeneticDiversityInAkitas.php. Accessed 10 April 2017.
